# Sport mental health continuum in young Brazilian athletes: adaptation and psychometric properties

**DOI:** 10.1038/s41598-024-71752-1

**Published:** 2024-10-05

**Authors:** Willen Remon Tozetto, Fabrício João Milan, Cláudio Olivio Vilela Lima, Michel Milistetd

**Affiliations:** Sport Pedagogy Research Center, Federal University of Santa Catarina, R. Dep. Antônio Edu Vieira - Pantanal, Florianópolis, SC Brazil

**Keywords:** Adolescent health, Psychological well-being, Psychometric study, Statistical factor analyses, Youth sports, Quality of life, Population screening, Psychology and behaviour, Human behaviour

## Abstract

Instruments used to assess the mental well-being of young athletes in Brazil are scarce. Therefore, the present study aimed to translate, cross-culturally adapt for young athletes, and gather evidence of validity for the Sport Mental Health—Short Form (S-MHC) for use in Brazilian Portuguese. The research was conducted in five stages: translation, synthesis, back-translation, expert review, and validation of the psychometric properties. For validation, 246 young athletes of both genders (88 females, 35.8%), aged between 12 and 18 years (14.5 ± 1.9 years), were recruited. Psychometric methods were employed to confirm and validate the translated and adapted versions of the S-MHC for young athletes, including internal consistency using Cronbach's alpha and McDonald's omega, composite reliability, Item Characteristic Curve (ICC) using Item Response Theory (IRT), and Confirmatory Factor Analysis (CFA). Two structures were tested, with Model 1 loading the 14 items of the translated version of the S-MHC into a single latent factor and Model 2 loading the items into three factors related to emotional, social, and psychological sport well-being. Both models showed good validity, consistency, and reliability measures and can be used to investigate the sport well-being of young athletes. It was concluded that the translated version of the S-MHC in Brazilian Portuguese can be used to assess the sport well-being of young athletes in Brazil. Model 2 structure is recommended to observe the different nuances of emotional, social, and psychological well-being.

## Introduction

Mental health can be operationalized through the comprehensive model of mental health, which acknowledges the existence of two opposing and distinct concepts: mental well-being and mental illness. It’s important to note that these two concepts are not mutually exclusive, meaning an individual may experience different levels of mental well-being while dealing with a mental illness. In general, mental well-being reflects a state in which individuals can utilize their own abilities in everyday situations^1–3^. For example, they are able to cope with the normal stress of life, work productively and fruitfully, or contribute to their community^1–3^. On the other hand, mental illness is defined as "a behavioral or psychological syndrome or pattern that occurs in an individual"^4^ affecting perceptions, relationships, and interactions including disorders such as anxiety, depression, and psychotic disorders. That for, Keyes asserts that the feeling of high well-being is as important for individuals with disorders as for those without health conditions^1–3^. As an increasingly prominent characteristic in society, ‘feeling good’ has become a major concern for young people because of generational changes, becoming a necessary condition for complete health^1–3^. In this context, sport emerges as a powerful tool to promote mental well-being.

Sports has been the subject of numerous studies, given that the sports environment enables human development with the potential for transformation throughout the lifespan and for providing well-being, in addition to being an exercise practice^5–7^. Specifically in adolescence, positively structured and guided sports can contribute to young people’s development^8^. Similarly, sports presents both physical and psychological challenges that require a careful balance to ensure positive transformation^6^. Otherwise, it will be negative. As an example, adolescent athletes' concerns include unique stressors such as rigorous training, stereotypical prejudices about academic and social capabilities, pressure from parents and coaches, and a shift from social identity to an athletic identity^9^. These experiences have an impact on sleep quality, recovery time from training, nutrition, motivation to practice, among others, which negatively modulate mental well-being^9^.

Despite the potential of sport to promote well-being, research in the sports field tends to seek answers regarding the frequency and prevention of mental disorders. In a recent scoping review^10^, only seven out of seventy-eight studies analyzed the observed positive psychological aspects. Among these studies, only one used an instrument aimed at assessing well-being, which hinders comprehensive evaluation of the construct in these environments^11^. The instrument adopted in the study mentioned in the review was the Mental Health Continuum—Short Form^12,13^. This instrument, devised by Corey Keyes, is one of the few questionnaires available to assess mental well-being^10^. Currently, this instrument has been translated and adapted for use in various countries, including Brazilian Portuguese^14^ and adolescents in Portugal^15^. However, the generic nature of the instrument presents itself as a limiting factor for its use as it does not specify a target audience, either in terms of populations or specific contexts. Recognizing this need, particularly within the sports setting, Foster and Chow^16^ developed an adaptation of the instrument, resulting in the Sport Mental Health Continuum—Short Form (Sport MHC-SF). The authors proceeded from the premise that athletes with higher well-being are more likely to thrive in a competitive environment, as sports demands and barriers can lead to greater difficulty achieving high well-being^8,16^. This sport instrument has been used in recent years and has contributed to the understanding of the mental well-being in international sports landscape^5–7,17,18^ but it lacks evaluations in Brazil.

In recent years there has been an increase in mental health problems in the Brazilian population, with 19.2% of problems occurring in young people aged 19 or under^19^. Additionally, 58% of adolescents engage in sports,^20^, with great potential for change to impact well-being. There is an emerging demand for research on athletes' well-being in order to establish policies to increase opportunities for practices aimed at safe sports, athletes support, mental health literacy and well-being promotion in Brazil. Thus, translating the Sport MHC-SF into Portuguese and adapting it to the Brazilian context can significantly contribute to this scenario, assisting managers, coaches, physical education teachers, and sports psychologists in assessing the sports well-being of young athletes.

Therefore, this study aimed to translate, cross-culturally adapt for young athletes, and gather evidence of validity for the Sport Mental Health—Short Form for use in Brazilian Portuguese. Among the validation search processes, the instrument will be analyzed under two distinct structures. The first approach employs a unifactorial structure to investigate the latent general factor of sports well-being. This structure is commonly employed to seek validation evidence in instrument studies. In contrast, the second approach adopts a second-order multifactorial structure, based on the three domains of well-being initially proposed by Keyes and adapted for the sports context by Foster and Chow. This multifactorial approach allows for a more specific analysis of the constructs involved (emotional, social, and psychological), offering new insights and potential intervention strategies in the context of sports well-being.

## Methods

### Study design

This study aimed to translate, cross-culturally adapt, and gather evidence of validity for the Sport—Mental Health Continuum (S-MHC) instrument^16^ for adolescent athletes in Brazilian Portuguese. Five separate phases were carried out in two stages of the cross-cultural adaptation process suggested by Beaton et al.^21^, which are: Stage 1: (1) Translation; (2) Synthesis; (3) Back-translation; (4) Expert review; and Stage 2: (5) Investigation of psychometric properties. Additionally, the ITC Guidelines for Translating and Adapting Tests (Second Edition)^22^ were followed.

In the first phase, the translation was conducted with two native speakers of Brazilian Portuguese. The second phase was led by the first author of this study, who synthesized the translated instruments from the first phase. The third phase was conducted by two other Brazilian natives and compared to the original version of Foster and Chow^16^. In the fourth phase, the synthesized instrument was sent to doctoral university professors for content validation and adapted as needed. In the fifth and final phases, cross-sectional data were collected from one of the country’s main sports clubs. Young people of both genders, aged 12–18 years, practicing various sports were invited. All ethical procedures were performed. Data were collected through an online form containing the revised questionnaire from the fourth phase as well as sociodemographic and sports data. Various psychometric tests including internal consistency, composite reliability, confirmatory factor analysis, and Item Response Theory were performed to confirm the structure and validity of the translated and adapted instruments.

### Stage 1—translation and adaptation

#### Phase 1—translation

The original version of the questionnaire was translated into Brazilian Portuguese by two native Portuguese speakers, fluent in English. Both were Ph.D. students in Physical Education, one focusing on sports research and the other on health research. One of the translators had previous exposure to the instrument and was familiar with the translation process adopted by Foster and Chow, "… with sport simply substituted for life or society"^16^ from the original questionnaire. Each translator worked independently and generated two translation files.

#### Phase 2—synthesis

The two translated versions in Phase 1 were forwarded to the first author of this study for compilation into a single version. The author retained the considerations made by the translators and contacted them in the case of discrepancies. In case of doubts and questions, other scales of the same instrument were applied. Among the scales used as aids for translation were (I) the original scale of the Mental Health Continuum (MHC) developed by Keyes^13^; (II) Translation and adaptation for adolescents in Portuguese from Portugal of the MHC by Matos et al.^15^; and (III) Translation and adaptation for Portuguese from Brazil of the MHC^14^. It is worth noting that neither instrument has the sports specificity of the instrument adapted by Foster and Chow^16^ and, therefore, it was used as an aid for translation, adaptation, and synthesis. Subsequently, the synthesized version was sent for a new round of approval from the translators. In this phase, it was decided to call the instrument in Portuguese “Contínuo de Saúde Mental no Esporte – Versão resumida” (CSME).

#### Phase 3—back-translation

This phase involved backward translation of the version achieved in Phase 2, returning to the English language by a third Ph.D. student in Physical Education. After back-translation, a fourth Ph.D. student in Physical Education conducted a semantic comparison with the CSME instrument to form an English version. The two students were fluent in English. At this point, it was decided to follow the format used by Matos et al.^15^, adding small explanations within parentheses to facilitate young participants’ understanding. For example, items 4, 5, 6, and 11 received an explanation about who is part of the "sports community," including "teammates, coaches, parents, managers, and other sports professionals." A new version of the instrument was obtained during this phase and sent to specialists.

#### Phase 4—expert review

Five university professors with PhDs in Physical Education, specializing in health (N = 1), psychologic and health (N = 1) and sports (N = 3) fields, were invited to evaluate the questionnaire's content. An invitation letter was sent, and upon the professors' consent, documents for analysis were dispatched. The documents included: I—General information about the instrument; II—Instructions and form for content validation; III—Translated version of the CSME in Phase 3; and IV—Analytical matrix of the instrument. These professionals rated the content in three domains: language clarity, practical relevance, and theoretical significance^23^. Each domain featured on a 5-point Likert scale ranging from: Inadequate (1), Somewhat adequate (2), Acceptable (3), Adequate (4), and Very adequate (5). In addition, there was a specific field for contributions and guidance written by professionals.

To achieve acceptable levels among the experts for each questionnaire item, the Content Validity Coefficient (CVC) was calculated for each of the three content domains^23^. CVC corresponds to the calculation of the mean scores of the experts divided by the maximum possible score minus bias. The bias risk was calculated as follows: Bias risk = (1/number of judges) number of judges or Bias risk = (1/5)5. This risk corresponded to a value of 0.00032. Based on the experts' scores, the mean for each domain was calculated and divided by the number of judges (N = 5), and the bias risk was subtracted. Values below 0.8 were considered unacceptable and subjected to changes, while CVC values ≥ 0.80 were deemed acceptable. Values below the cutoff point in any domain underwent restructuring according to the experts' contributions and guidance, and were then resubmitted for a second round of evaluation. The values were calculated in an excel spreadsheet.

### Stage 2—investigation of psychometric properties

#### Sample

The study was conveniently conducted at a sports club of excellence in training of young Brazilian athletes located in the Belo Horizonte/MG region, which houses eight sports modalities: futsal, volleyball, basketball, swimming, judo, tennis, artistic gymnastics, and trampoline gymnastics. In addition, as one of the leading centres for athlete development, the club attracts talent from various regions across the country, reflecting geographical diversity in its team composition. Additionally, the institution welcomes athletes from different socioeconomic backgrounds, encompassing classes A, B, and C. This socioeconomic and regional plurality contributes to the cultural richness and diversity of experiences among the athletes, which may be a significant factor in the composition of this sample.

In the initial contact, the study's aim was explained, assessment was illustrated, and the evaluation period was defined. The research partnership was established through a declaration of consent and authorization from the institution to conduct the project. Data collection were set between July and December 2023.

Researchers emailed participants (athletes and parents for minors' consent) with corresponding forms. Each form included pertinent information for the project, sociodemographic questions (Gender; Age; Affiliation), sports-related inquiries (Modality; Competitive Level; Training Frequency), and the adapted CSME questionnaire. All forms were completed online. Eligibility criteria included being aged between 12 and 18 years, enrolled as an athlete at the institution, and having participated in training sessions for at least six months.

A total of 611 emails were sent corresponding to the number of enrolled athletes within the specified age range at the institution. A total of 253 responses were obtained, however, seven athletes were excluded for reporting ages outside the required range. The final sample consisted of 246 athletes from various sports disciplines, representing 40.3% of the club's athletes.

#### The sport mental health continuum instrument

S-MHC is a self-report questionnaire that does not require the presence of an evaluator. The instrument comprises 14 items related to emotional well-being (EWB; N = 3), social well-being (SWB; N = 5) and psychological well-being (PWB; N = 6) each with six possible responses on a Likert type scale in increasing intensity, as follows: (0) Never; (1) Once or twice; (2) About once a week; (3) About 2 or 3 times a week; (4) Almost every day; (5) Every day. All items were formulated positively, making it unnecessary to apply reverse scoring to any item. Before responding to the items, participants are instructed in the header to reflect on how they have felt during their participation in sports over the past month. Subsequently, all 14 items are answered based on the following premise: “During the last month, how often has your participation in sports made you feel…” followed by items such as: Interested in your sport (EWB); That people in your sport, in general, are nice to others (SWB); That you had sports experiences that challenged you to grow, develop, and become a better person (PWB).

#### Research ethics committee

The present study was submitted to the research ethics committee of the Federal University of Santa Catarina (CEPSH-UFSC) and obtained a favorable opinion under number 5,972,228. All ethical procedures outlined in Resolution 466/12 of June 12, 2012, by the National Health Council, and in the Helsinki Declaration, were followed. The online form presented a Free and Informed Assent Form to the young participants and a Free and Informed Consent Form to their parents or guardians of these young participants. Additionally, consent was obtained by email from the authors of the sports version (Brian J. Foster and Graig M. Chow) and the original version of the instrument (Corey Keyes), authorizing the translation and adaptation process.

#### Data analysis

Data were analyzed using RStudio software version 2021.09.0. When necessary, the “*lavaan*” package was used for Confirmatory Factor Analysis (CFA). For Cronbach's alpha coefficient and McDonald's omega coefficient, JASP statistical software version 0.16.4 was used. As descriptive statistics, absolute and relative frequencies, means and standard deviations were used. Mean values of the total CSME was presented according to the categories of gender (male; female); age (younger; older), affiliation (member; non-member); modality (collective; individual); competitive level (municipal; regional; national; international) and training frequency (1 to 4 days/week; 5 days/week; 6 to 7 days/week). Independent t-tests were used to compare variables with two categories and one-way ANOVA for the others. The critical value of 5% was used for statistical significance.

The asymmetry and kurtosis of the data were observed, and the normal distribution of the data will be tested using the Shapiro–Wilk test for each of the 14 items and the overall sum of the CSME. Normative values of ± 3 for asymmetry and ± 10 for kurtosis were considered^24^. Non-significant *p*-values indicate normality of the data. Values within the normative values were expected, but without a normal distribution due to the ordinal type of Likert-type responses.

#### Confirmatory factor analysis

Owing to the ordinal nature of the data, the Weighted Least Squares Mean and Variance adjusted (WLSMV) estimation method was used for Confirmatory Factor Analysis (CFA)^24^. Following Foster and Chow's^16^ choice to conduct CFA instead of exploratory factor analysis, the MHC-SF's factor structure has been highly validated by Keyes^13^ and subsequent adaptations^14,15^.

CFA was conducted using two models to confirm the factorial structure of CSME. These models followed those used by Foster and Chow^16^ and the Portuguese-translated version of the MHC^14^. "Model 1" consisted of a single factor loading the 14 items representing overall well-being. "Model 2" comprised three factors: items 1 to 3 related to emotional sports well-being, items 4 to 8 related to social sports well-being, and items 9 to 14 related to psychological sports well-being. Furthermore, these three factors were loaded onto a second-order general factor. In both cases, bootstrapping was performed for thousand resampling to estimate the robust 95% confidence intervals (CI) for the factor loadings. The factor loadings were considered as follows: 0.3 low loading; from 0.3 to 0.45 medium loading; from 0.5 to 0.79 high loading; and above 0.8 very high loading.

The fit indices of the considered models included the following criteria and cutoff points: degrees of freedom (df) equal to or greater than 1, *p*-value of the chi-square test (χ^2^) above 0.05, χ^2^/df ratio less than 3.00. Comparative Fit Index (CFI) and Tucker–Lewis Index (TLI) above 0.90 indicating reasonable fit and above 0.95 indicating good fit. Root Mean Square Error of Approximation (RMSEA) and Standardized Root Mean Square Residual (SRMR) below 0.10 indicating reasonable fit and below 0.08 indicating good fit^24^. Modification indices (MI) for residual covariances were also considered, with a threshold of 6.8 as the cutoff point. The polychoric correlations obtained using the WLSMV estimator are reported in Supplementary Material 1.

#### Internal consistency

The Cronbach's alpha coefficient was used to assess the internal consistency of the measurement instrument. The interpretation ranges for Cronbach's alpha coefficient are widely recognized. Values less than 0.60 are considered inadequate. Coefficients in the range of 0.60–0.69 are viewed as acceptable, while values between 0.70 and 0.79 are considered good. Coefficients of 0.80 or higher are interpreted as indicative of very good internal consistency. Due to recent criticisms of Cronbach's alpha regarding its limitations as a measure of internal consistency reliability, such as sensitivity to the number of scale points and assumptions of tau-equivalence^25^, we also computed McDonald's omega. McDonald's omega provides a more robust estimate by considering the variance explained by the factor loadings of each item, thus offering a potentially more accurate reflection of the internal consistency of a measurement instrument^25^. The same interpretation of values of alpha is used.

#### Composite reliability

The composite reliability is a crucial measure that indicates the degree to which the item loadings of a measurement instrument accurately reflect the underlying latent constructs or factors. Unlike Cronbach's alpha, which is a measure of internal consistency, composite reliability considers the factor loadings of each item in the measurement model. It provides a more refined estimate of reliability, especially in models with multiple indicators per latent variable^25^. The same interpretation of values of alpha and omega are used.

#### Item response theory

Item Response Theory (IRT) was employed to model how participants' latent response ability correlate with the likelihood of answering items correctly^26^. Response shift levels were observed to detect potential variations in participants' response patterns across different conditions. Considering the six response possibilities on the Likert-type scale of the CSME questionnaire, five change thresholds are observed (t1, t2, t3, t4, and t5). For instance, shift t1 represents a change from “1—Never” to “2—Once or twice a month,” and so forth. Threshold values indicate participants' ability to respond correctly across these five points, ranging from ± 3. When observed, the increasing or decreasing linearity of response thresholds will be considered adequate^26^. Any deviation from thresholds linearity may indicate violations of IRT assumptions^26^.

## Results

### Stage 1—translation and adaptation

The instrument of the “Contínuo de Saúde Mental no Esporte – versão resumida” (CSME) has been satisfactorily translated and adapted for use in the Portuguese language. The final version of the instrument can be viewed in supplementary material 2. A back-translation of the document was conducted and compared with the original version. The differences between the versions do not alter the content of the instrument; rather, they serve to localize the information for the Brazilian culture and language. Subsequently, the instrument was submitted for content validation by five professors. The information is presented below.

Table 1 presents the coefficients obtained for each round of content validation. In the first version, 5 items (questions 3, 7, 8, 9, and 11, specifically) showed CVC below 0.80 for language clarity. Additionally, Item 11 also exhibited a CVC below for pertinence and relevance. In these cases, the items were restructured for the second round. All the items were deemed suitable in the second round.

**Table 1:** Rating assigned by expert professionals to the content domains of the translated version of CSME.

Items	CVC					
	1st round			2nd round		
	LC	PP	TR	LC	PP	TR
CSME—1	0.88	1.00	1.00	–	–	–
CSME—2	1.00	1.00	0.88	–	–	–
CSME—3	0.76*	0.92	0.92	0.96	1.00	1.00
CSME—4	0.80	0.92	0.92	–	–	–
CSME—5	0.88	1.00	1.00	–	–	–
CSME—6	0.92	0.96	0.96	–	–	–
CSME—7	0.72*	0.92	0.92	0.96	0.96	0.96
CSME—8	0.56*	0.79*	0.79*	0.92	0.92	0.92
CSME—9	0.68*	0.92	0.84	0.92	1.00	1.00
CSME—10	0.96	1.00	1.00	–	–	–
CSME—11	0.68*	0.96	0.96	0.96	1.00	1.00
CSME—12	0.96	1.00	1.00	–	–	–
CSME—13	1.00	1.00	1.00	–	–	–
CSME—14	0.88	1.00	1.00	–	–	–

*CVC* content validity coefficient, *LC* language clarity, *PP* practical pertinence, *TR* theoretical relevance, *: CVC < 0.80.

### Stage 2—investigation of psychometric properties

#### Phase 5—investigation of psychometric properties

The sample consisted of 246 athletes, with 88 (35.8%) females, and the mean age was 14.5 ± 1.9 years, ranging from 12 to 18 years old. The sports practiced by the adolescents were as follows: 66 (26.8%) futsal, 60 (24.4%) volleyball, 38 (15.4%) swimming, 28 (11.4%) tennis, 20 (8.1%) judo, 19 (7.7%) basketball, 13 (5.3%) artistic gymnastics, and two (0.8%) trampoline gymnastics. In Table 2, sociodemographic and sports characteristics are presented in comparison to the values obtained from the CSME instrument.

**Table 2:** Sociodemographic characteristics according to the average CSME of Brazilian adolescent athletes.

Item	CSME ($\overline{\text{x} }$ ± SD)	*p*-value
Gender		0.179
Male (n = 158)	61.7 ± 6.25	
Female (n = 88)	60.4 ± 7.42	
Age		0.047*
Younger (12–14) (n = 138)	61.6 ± 6.58	
Older (15–18) (n = 108)	59.8 ± 7.49	
Affiliation		0.078
Member (n = 177)	60.4 ± 7.18	
Non-Member (n = 69)	62.1 ± 6.53	
Modality		0.011*
Team (n = 145)	61.8 ± 6.18	
Individual (n = 101)	59.5 ± 7.95	
Competitive Level §		0.068
Municipal (n = 11)	58.5 ± 9.22	
Regional (n = 92)	62.3 ± 6.02	
National (n = 114)	59.9 ± 7.65	
International (n = 29)	60.9 ± 6.01	
Training Frequency §		0.118
1 to 4 days/week (n = 27)	58.2 ± 9.81	
5 days/week (n = 114)	61.3 ± 6.54	
6 to 7 days/week (n = 105)	61.1 ± 6.64	

*SD* standard deviation, *: *p*-value ≤ 0.05, §: analysis from one-way ANOVA.

Significant differences were observed for age (*p* = 0.047), with older athletes showing lower mental well-being (diff = 1.8). Furthermore, athletes in team sports exhibited greater well-being compared to their counterparts in individual sports (diff = 2.3; *p* = 0.011). The remaining variables did not show significant differences.

The Shapiro–Wilk test confirming a skewed distribution of the data (W(246) = 0.494–0.813, *p* < 0.001), similar to the original sports scale^16^. Specifically, Item 2 of the CSME exhibits Skewness and Kurtosis above the cutoff points^24^. Further characteristics are presented in Table 3.

**Table 3:** Descriptive values of CSME responses by adolescents.

Item	$\overline{\text{x} }$± SD	SE	Skewness	Kurtosis	Shapiro–wilk (w)
CSME—1	4.05 ± 0.68	0.04	− 1.10	5.02	0.752*
CSME—2	4.65 ± 0.78	0.05	− 3.25	12.57	0.494*
CSME—3	4.07 ± 1.09	0.07	− 1.36	1.89	0.791*
CSME—4	3.95 ± 0.99	0.06	− 1.24	1.95	0.813*
CSME—5	4.58 ± 0.75	0.05	− 2.43	7.62	0.594*
CSME—6	4.54 ± 0.83	0.05	− 2.63	9.04	0.594*
CSME—7	4.28 ± 0.84	0.05	− 1.73	5.21	0.741*
CSME—8	4.49 ± 0.83	0.05	− 2.46	8.38	0.628*
CSME—9	4.08 ± 0.87	0.06	− 1.57	4.45	0.766*
CSME—10	4.35 ± 0.80	0.05	− 1.64	4.42	0.733*
CSME—11	4.61 ± 0.68	0.04	− 2.45	9.58	0.599*
CSME—12	4.57 ± 0.67	0.04	− 1.83	4.47	0.651*
CSME—13	4.05 ± 0.99	0.06	− 1.29	2.02	0.805*
CSME—14	4.59 ± 0.79	0.05	− 2.55	8.06	0.579*
Sum	60.8 ± 7.04	0.45	− 1.50	3.41	0.892*

$\overline{\text{x} }$: mean, *SD* standard deviation, *SE* standard error, *: *p*-value ≤ 0.05.

#### Confirmatory factor analysis

The CFA was performed, and the adequacy criteria and modifications are presented in Table 4.

**Table 4:** Fit indices for the two factor structure models of the CSME.

	df	χ^2^	χ^2^ /df	CFI	TLI	RMSEA (IC95%)	SRMR
Model 1	77	229.806*	2.98	0.940	0.929	0.090 (0.077; 0.104)	0.076
Model 1 ajusted	74	153.468*	2.07	0.969	0.962	0.066 (0.051; 0.081)	0.098
Model 2	74	211.404*	2.86	0.946	0.934	0.087 (0.073; 0.101)	0.072
Model 2 ajusted	71	140.637*	1.98	0.973	0.965	0.063 (0.048; 0.079)	0.060

*df* degrees of freedom, χ^2^: chi-square test, *TLI* Tucker–Lewis index, *CFI* comparative index, *RMSEA* root mean square error of approximation, *SRMR* standardized root mean square residual, *: *p* < 0.001.

All fit index values appeared appropriate according to the theoretical assumptions, except for the *p*-value of χ^2^. It is worth noting that χ^2^ analysis is sensitive to small discrepancies between the theoretical model and observed data and often appears inappropriate^24^. However, the other fit indices that were considered were satisfactory. Model 1 showed a slight visual difference from Model 2, indicating that both models fit satisfactorily and similarly. In relation to the modification indices, a residual correlation was added for the same items in Models 1 and 2, between Items 2 and 14, 6 and 7, and 9 and 10.

Table 5 lists the factor loadings for the models. We chose to present the CFA factor loadings of the two models without modification indices because of how the scale will be used by other researchers, maintaining the "usual" structure of the questionnaire. In general, the factor loadings were high and very high, ranging from 0.518 to 0.818, indicating a substantial and robust relationship between latent constructs and their observed indicators. Comparatively, Model 2 showed higher factor loadings and better fit indices than Model 1. However, these differences are too small to be considered relevant and decisive when selecting an ideal model.

**Table 5:** Factor Loadings and 95% Confidence Intervals for the CSME Models.

	Factor loading			
	Item	Model 1	Model 2	Dif
EWB	CSME—1	0.583 (0.473; 0.695)	0.650 (0.505; 0.762)	0.067
	CSME—2	0.518 (0.383; 0.679)	0.570 (0.415; 0.733)	0.052
	CSME—3	0.618 (0.551; 0.711)	0.694 (0.595; 0.783)	0.076
SWB	CSME—4	0.597 (0.499; 0.690)	0.618 (0.522; 0.713)	0.021
	CSME—5	0.745 (0.654; 0.842)	0.767 (0.680; 0.865)	0.022
	CSME—6	0.790 (0.701; 0.861)	0.818 (0.729; 0.889)	0.028
	CSME—7	0.529 (0.386; 0.637)	0.543 (0.396; 0.653)	0.014
	CSME—8	0.782 (0.719; 0.863)	0.809 (0.749; 0.892)	0.027
PWB	CSME—9	0.661 (0.593; 0.773)	0.673 (0.603; 0.784)	0.012
	CSME—10	0.676 (0.603; 0.786)	0.685 (0.611; 0.793)	0.009
	CSME—11	0.678 (0.580; 0.753)	0.689 (0.585; 0.766)	0.011
	CSME—12	0.684 (0.570; 0.772)	0.694 (0.583; 0.783)	0.010
	CSME—13	0.711 (0.648; 0.796)	0.722 (0.661; 0.805)	0.011
	CSME—14	0.661 (0.580; 0.787)	0.669 (0.587; 0.800)	0.008

*EWB* Emotional Well-Being, *SWB* Social Well-Being, *PWB* psychological Well-Being, *Dif.* difference.

**Fig. 1:**
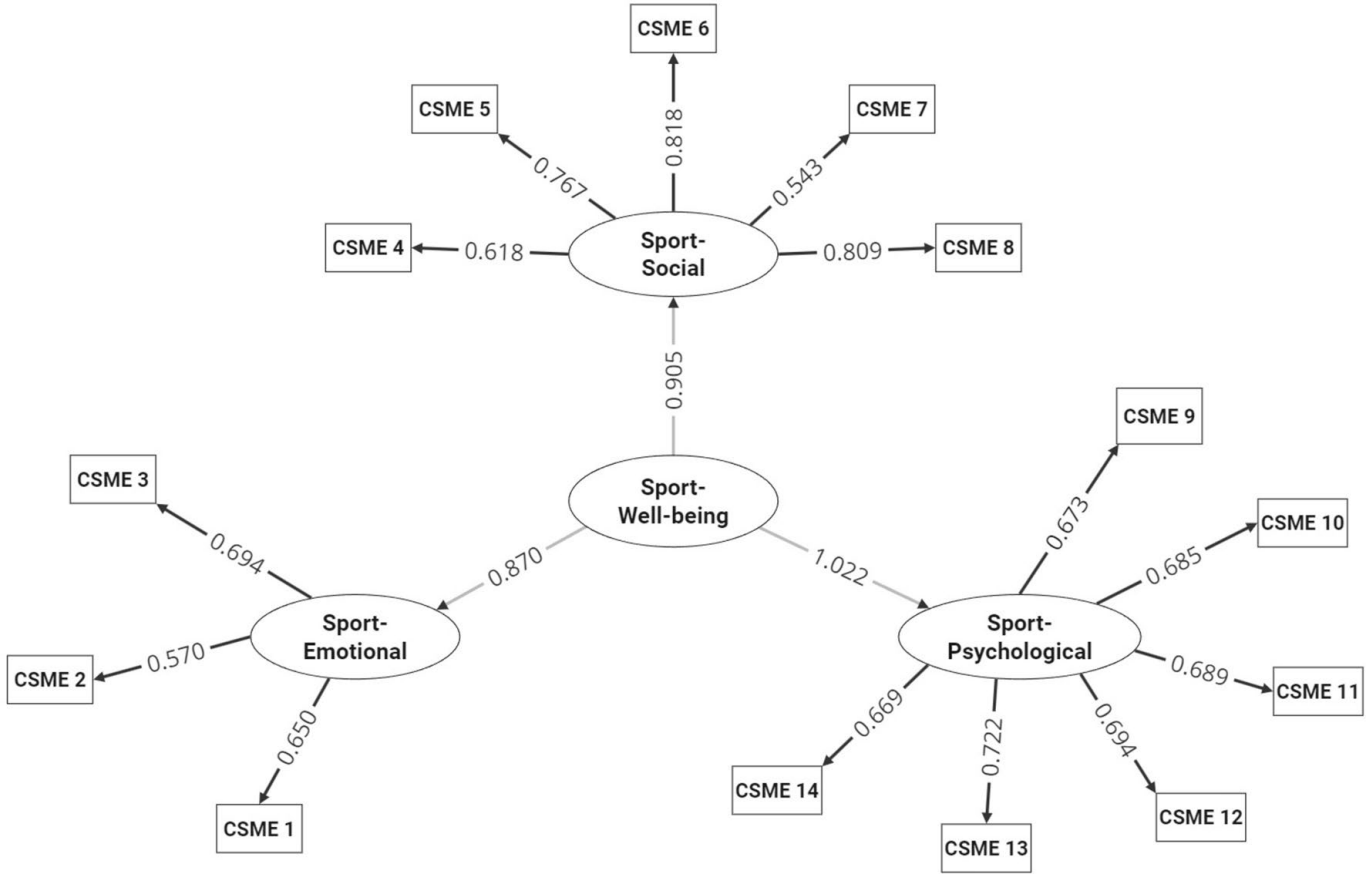
Graphical representation of confirmatory factor analysis following model 2. Source: Developed by the authors (2024).

Since the differences between the models were minimal, Model 2 was chosen for the graphical representation. This representation is illustrated in Fig. 1.

#### Internal consistency

The reliability analysis results revealed a robust Cronbach’s alpha coefficient of 0.87 (95% CI 0.84; 0.89) for the model 1. This value indicates high internal consistency among the variables, suggesting that the instrument has good reliability. Moreover, the 95% confidence interval underscores the precision of this estimate (> 0.80) and suggests a robust internal consistency. For Model 2, it was 0.512 (95% CI 0.406; 0.602), 0.741 (95% CI 0.685; 0.789), and 0.763 (95% CI 0.715; 0.805) for emotional, social, and psychological well-being considering the constructs, respectively. Therefore, the emotional construct presents inadequate consistency, while the others present good consistency.

Regarding McDonald's omega coefficient, an omega of 0.89 (95% CI 0.86; 0.91) was found, corresponding to high internal consistency too. For the Model 2, considering the emotional, social, and psychological well-being constructs respectively, it was 0.620 (95% CI 0.461; 0.778), 0.751 (95% CI 0.703; 0.800), and 0.770 (95% CI 0.725; 0.815). Thus, the emotional construct presents acceptable consistency and good consistency for social and psychological.

#### Composite reliability

The composite reliability was 0.916 for Model 1 and is considered indicative of high internal consistency. For Model 2, considering the constructs, it was 0.674, 0.840, and 0.844 for emotional, social, and psychological well-being, respectively. For emotional well-being, a composite reliability of 0.674 indicated acceptable internal consistency. The other constructs exhibited high internal consistency.

#### Item response theory

**Fig. 2:**
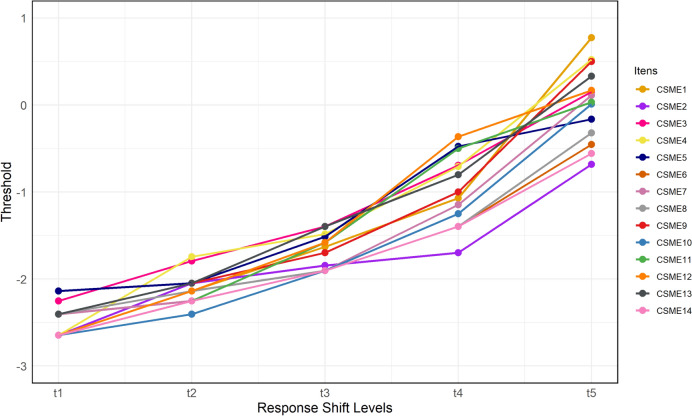
Graphical analysis of CSME item thresholds by response shift level. Source: Developed by the authors (2024).

The thresholds showed growing linearity of response, indicating that the test items were in line with the theoretical expectations of IRT^26^. That is, as participants' latent response ability increases, the probability of responding correctly to the item also increases consistently^26^ (see Fig. 2).

## Discussion

This study aimed to translate, cross-culturally adapt for young athletes, and gather evidence of validity for the Sport Mental Health—Short Form for use in the Brazilian Portuguese language. The translation, back-translation, and synthesis processes involved five native Portuguese speakers who were fluent in English, yielding satisfactory results. The translated instrument was scrutinized by specialists and remained appropriate in terms of language clarity, practical relevance, and theoretical significance. Various psychometric analyses were conducted, including internal consistency, CFA, composite reliability, and IRT, all of which yielded satisfactory results.

Skewness and kurtosis above the cutoff points were observed for Item 2 of the CSME^24^, attributed to a high concentration of responses at option 5 on the Likert-type scale. This item assesses how frequently athletes feel 'Interested in their sport,' reflecting motivational aspects crucial for emotional well-being. This data discrepancy may be influenced by the sampling location, a reference point for sports practice in the region. However, this does not necessarily indicate an issue with the item but rather a characteristic of the study location. Consequently, the item was retained in the instrument after subsequent tests showed no further issues. Nevertheless, caution is advised when using this item in future applications of the instrument. If it exhibits problems in new validations, it may warrant reconsideration. The original version by Foster and Chow^16^ and its subsequent uses^5–7^ did not exhibit such characteristics, underscoring the influence of sampling location with distinct features.

The values corresponding to the internal consistency of the instrument showed indices indicating robust internal consistency (alpha = 0.87; omega = 0.89). Foster and Chow^16^ found similar Cronbach's alpha values, corresponding to 0.89, but did not conduct the omega test. Similarly, Matos et al.^15^ reported an alpha of 0.90. Machado and Bandeira^14^ reported both values (alpha = 0.96; omega = 0.93), indicating a good internal structure. Recent studies utilizing the English version of the S-MHC reported alpha values with high reliability levels^5–7^. All these results showed better internal consistency than those found by Keyes in the validation of the original version of the document (alpha = 0.74)^12^.

Owing to the issues presented by Cronbach's alpha, in addition to McDonald's omega, composite reliability was calculated for both models^27^. Model 1 showed good composite reliability, while Model 2 exhibited moderate composite reliability for emotional well-being and good for social and psychological well-being. Few studies have presented composite reliability in the MHC structure, with the Brazilian Portuguese version being one of them^14^ but none in the S-MHC structure. The authors reported high composite reliability for Model 1 but low composite reliability for the model with the three constructs^14^. Together with the internal consistency values, these results demonstrate that the CSME in Portuguese presents good indices for use in young athletes across its different presented models.

The results of the CFA in this study demonstrated that the instrument can be utilized in a single-factor model (Model 1). This result was unexpected, considering that the instruments tested by Keyes^13^ and their adaptations mostly present a three-factor structure^12,28,29^. The same was found for Foster and Chow's sports instrument^16^, in which only the structure corresponding to Model 2 showed adequate indices. In this case, it is noteworthy that in Model 1, the RMSEA, TLI, and CFI in Foster and Chow’s^16^ study were close to the cutoff points, and cultural and generational differences may explain the slight variation in models. Machado and Bandeira^14^ found different results, with both models being adequate, and the unidimensional model exhibiting greater explanatory power for the items. This demonstrates that, for young Brazilian athletes, it is possible to use a single structure to understand mental well-being.

Despite these results demonstrating the versatility of the instrument across its models, it is important to emphasize that a three-factor structure contributes to a detailed understanding of the latent structure of the phenomenon, which facilitates decision-making for evaluators and is strongly recommended by specialized literature^12^. It is necessary for other studies to retest the models to confirm their use, whether in their structure with a single latent construct or three, as other studies with the MHC scale in adolescents do not corroborate the findings of the present study. For example, in French-speaking Canadian adolescents^28^, the opposite was observed, with the three-factor structure presenting better indices, whereas the one-factor structure did not present adequate indices.

Additional analyses should be conducted to confirm the structure of the instrument and its validity for young athletes in Brazil. These analyses include convergent and divergent validity, item fit to the Item Response Theory Rating Scale model, network analysis, and additional models, such as a bi-factor model. It is necessary to test for possible discrepancies between groups, such as differences between genders and ages, socioeconomic classes, and training locations, including different sports or different sporting characteristics, such as team versus individual, technical-combinatorial versus invasion versus marksmanship/precision versus combat sports.

This study had some limitations. Data collection was conducted online, which may have resulted in bias in responses, since adolescents could respond at any time of the day. Furthermore, young athletes may fear being classified as having poor well-being, which could be reflected in their responses. However, adolescents were assured that guardians, such as family members, coaches, and other participants in the sports context, would not be informed of individual research results, and their respective names were replaced with numbers to ensure the confidentiality of information, following the General Data Protection Law of Brazil^30^.

Another significant limitation concerns the representativeness of the sample. Although we achieved a representative sample of the study unit (40.3% of the total), it is important to note that the city is home to over 150,000 adolescents aged 12–18 years. Considering that approximately 30% of youth engage in some form of sports activity in the city (a percentage lower than the current estimate of 58%^20^), the analysis of 611 athletes from the largest club in the region represents less than 1.5% of the total athletes. To achieve a 5% sample, approximately 2250 athletes would be needed in the city alone.

The National Household Sample Survey (PNAD) reports that 37.9% (61.3 million) of the total 161.8 million youth aged 15 to 17 years in Brazil participate in some form of sports activity^31^. Therefore, to represent 1% of this population, approximately 613,000 athletes distributed across the 26 states of the country would need to be analyzed. Due to the continental dimensions of Brazil, achieving numerically representative samples is a challenging task. Nevertheless, it is important to highlight that Confirmatory Factor Analysis (CFA), recognized for requiring a substantial sample size, demonstrated excellent fit indices. It is generally recommended to have a minimum of 10 participants per item for factor analyses^32^, a criterion that we exceeded, which should be adequate for initially exploring the psychometric properties of the CSME. An additional limitation of the study lies in the context analyzed, as the sports institution distinguish between members and nonmembers. This differentiation arises due to the nature of the institution, which operates as a private society but accepts athletes from outside. Such dynamics result in socioeconomic disparities between the groups and their potential implications. It is worth noting that no financial charges are imposed for participation in sports institutions’ training sessions, and in certain cases, non-member participants receive financial assistance, which could mitigate discrepancies among those involved.

## Conclusion

The translated and adapted version of the CSME for youth has been tested through various procedures and has proven to be valid and reliable in measuring the mental well-being of adolescent athletes, whether in its single- or three-latent-construct model. The possibility of using a specific instrument for evaluating the well-being of young athletes enables research directed towards this construct to be operationalized in the country.

It is recommended that the structure present in Model 2 be used so that a thorough assessment of the different constructs of well-being is presented, providing specific research and intervention possibilities regarding the dimensions of athletes. For researchers, it is recommended to present measures of internal validity (at least McDonald's omega and composite reliability) and conduct Confirmatory Factor Analysis of the instrument in their investigations.

## Supplementary Information

Supplementary Information 1.

Supplementary Information 2.

## Data Availability

The dataset comprises sensitive material from teenagers. Therefore, it is not available on freely accessible online platforms. In any case, researchers can provide data upon request to the corresponding author after signing an ethical responsibility agreement for the data. This information follows the General Data Protection Law of Brazil (Law No. 13,709, of August 14, 2018.

## Abbreviations

CEPSH-UFSC: Research Ethics Committee of the Federal University of Santa Catarina
CFA: Confirmatory Factor Analysis
CFI: Comparative Fit Index
CSME: Contínuo de Saúde Mental no Esporte – Versão resumida
CVC: Content Validity Coefficient
df: Degrees of freedom
IRT: Item Response Theory
MHC: Mental Health Continuum
MI: Modification indices
RMSEA: Root Mean Square Error of Approximation
SRMR: Standardized Root Mean Square Residual
S-MHC: Sport—Mental Health Continuum
TLI: Tucker–Lewis Index
WLSMV: Weighted Least Squares Mean and Variance adjusted
χ^2^: Chi-square test
