# Supplementary material for: Sport mental health continuum in young Brazilian athletes: adaptation and psychometric properties
Source: Sci Rep. 2024 Oct 5;14:23194. doi: 10.1038/s41598-024-71752-1 (PMC11455932; doi:10.1038/s41598-024-71752-1)
Supplement: Supplementary file 1 — Supplementary Information 1. [file 41598_2024_71752_MOESM1_ESM.docx]

| **Supplementary material 1**. CSME Inter-Item Polychoric Correlation Matrix. | | | | | | | | | | | | | | |  |  |
| --- | --- | --- | --- | --- | --- | --- | --- | --- | --- | --- | --- | --- | --- | --- | --- | --- |
| Itens | 1 | 2 | 3 | 4 | 5 | 6 | 7 | 8 | 9 | 10 | 11 | 12 | 13 | 14 | | |
| CSME - 1 | 1 | - | - | - | - | - | - | - | - | - | - | - | - | - | | |
| CSME - 2 | 0.265 | 1 | - | - | - | - | - | - | - | - | - | - | - | - | | |
| CSME - 3 | 0.495 | 0.361 | 1 | - | - | - | - | - | - | - | - | - | - | - | | |
| CSME - 4 | 0.281 | 0.156 | 0.453 | 1 | - | - | - | - | - | - | - | - | - | - | | |
| CSME - 5 | 0.324 | 0.300 | 0.326 | 0.434 | 1 | - | - | - | - | - | - | - | - | - | | |
| CSME - 6 | 0.539 | 0.417 | 0.409 | 0.370 | 0.669 | 1 | - | - | - | - | - | - | - | - | | |
| CSME - 7 | 0.335 | 0.098 | 0.257 | 0.266 | 0.370 | 0.544 | 1 | - | - | - | - | - | - | - | | |
| CSME - 8 | 0.418 | 0.279 | 0.458 | 0.459 | 0.680 | 0.665 | 0.434 | 1 | - | - | - | - | - | - | | |
| CSME - 9 | 0.458 | 0.349 | 0.456 | 0.395 | 0.383 | 0.374 | 0.330 | 0.467 | 1 | - | - | - | - | - | | |
| CSME - 10 | 0.298 | 0.461 | 0.483 | 0.471 | 0.430 | 0.400 | 0.271 | 0.384 | 0.671 | 1 | - | - | - | - | | |
| CSME - 11 | 0.432 | 0.255 | 0.388 | 0.381 | 0.615 | 0.563 | 0.361 | 0.547 | 0.347 | 0.324 | 1 | - | - | - | | |
| CSME - 12 | 0.260 | 0.330 | 0.440 | 0.493 | 0.473 | 0.516 | 0.291 | 0.602 | 0.420 | 0.457 | 0.498 | 1 | - | - | | |
| CSME - 13 | 0.288 | 0.286 | 0.395 | 0.502 | 0.626 | 0.525 | 0.420 | 0.560 | 0.447 | 0.394 | 0.529 | 0.584 | 1 | - | | |
| CSME - 14 | 0.433 | 0.530 | 0.387 | 0.359 | 0.526 | 0.451 | 0.170 | 0.587 | 0.448 | 0.482 | 0.378 | 0.469 | 0.450 | 1 | | |
| Sum | 0.620 | 0.572 | 0.720 | 0.664 | 0.733 | 0.786 | 0.590 | 0.777 | 0.712 | 0.692 | 0.709 | 0.706 | 0.745 | 0.699 | | |
| Note: Sum = Variable corresponding to the sum of CSME items. | | | | | | | | | | | | | | | |  |
