# Supplementary material for: Sport mental health continuum in young Brazilian athletes: adaptation and psychometric properties
Source: Sci Rep. 2024 Oct 5;14:23194. doi: 10.1038/s41598-024-71752-1 (PMC11455932; doi:10.1038/s41598-024-71752-1)
Supplement: Supplementary file 2 — Supplementary Information 2. [file 41598_2024_71752_MOESM2_ESM.docx]

**Supplementary material 2** - Portuguese version of the Sport Mental Health Continuum

| **Contínuo de Saúde Mental no Esporte – Versão resumida**  Por favor, responda às questões abaixo sobre como você tem se sentido durante sua participação no esporte. Marque a frequência semanal que melhor representa a maneira com que você se sentiu durante o último mês. | | | | | | | | | | | | |
| --- | --- | --- | --- | --- | --- | --- | --- | --- | --- | --- | --- | --- |
| 1  Nunca | | 2  Uma ou duas vezes no mês | 3  Cerca de uma vez por semana | 4  Cerca de duas ou três vezes por semana | 5  Quase todos os dias da semana | | | | 6  Todos os dias da semana | | | |
| **Durante o último mês, com que frequência a sua participação esportiva fez você se sentir...** | | | | | | | | | | | | |
| **Itens** | **Questões** | | | | | **1** | **2** | **3** | | **4** | **5** | **6** |
| 1 | Regularmente alegre, de bom humor, feliz, calmo e tranquilo no seu esporte | | | | |  |  |  | |  |  |  |
| 2 | Interessado(a) no seu esporte | | | | |  |  |  | |  |  |  |
| 3 | Satisfeito(a), contente e realizado(a) no seu esporte | | | | |  |  |  | |  |  |  |
| 4 | Que você teve algo importante para contribuir para a sua comunidade esportiva (colegas de equipe, treinadores, pais, gestores e outros profissionais do esporte) | | | | |  |  |  | |  |  |  |
| 5 | Que você pertence à uma comunidade esportiva (colegas de equipe, treinadores, pais, gestores e outros profissionais do esporte) | | | | |  |  |  | |  |  |  |
| 6 | Que a sua comunidade esportiva (colegas de equipe, treinadores, pais, gestores e outros profissionais do esporte) é um bom lugar para todos(as) os(as) participantes | | | | |  |  |  | |  |  |  |
| 7 | Que as pessoas no seu esporte, em geral, são legais com os outros | | | | |  |  |  | |  |  |  |
| 8 | Que o modo como o ambiente esportivo funciona faz sentido para você. | | | | |  |  |  | |  |  |  |
| 9 | Que você gostou da maioria dos seus comportamentos e atitudes no esporte | | | | |  |  |  | |  |  |  |
| 10 | Que você administrou bem as responsabilidades do dia a dia do seu esporte | | | | |  |  |  | |  |  |  |
| 11 | Que você teve relacionamentos de amizade, prazerosos e de confiança com as pessoas da comunidade esportiva (colegas de equipe, treinadores, pais, gestores e outros profissionais do esporte) | | | | |  |  |  | |  |  |  |
| 12 | Que você teve experiências esportivas que o(a) desafiaram a crescer, se desenvolver e se tornar uma pessoa melhor | | | | |  |  |  | |  |  |  |
| 13 | Que você foi confiante para pensar ou expressar suas próprias ideias e opiniões para as pessoas no seu esporte | | | | |  |  |  | |  |  |  |
| 14 | Que você tem um propósito, um objetivo, uma meta ou sentido dentro do seu esporte | | | | |  |  |  | |  |  |  |
